# E-health supported referral for patients with breast abnormalities at primary healthcare facilities in Ethiopia: protocol for a cluster-randomised controlled trial

**DOI:** 10.1186/s13063-025-09409-1

**Published:** 2026-01-10

**Authors:** Eyerusalem Getachew, Muluken Gizaw, Endale Anberber, Abel Shita, Alemnew Destaw, Sarah Sophie Rossner, Aragaw Tesfaw, Adamu Addissie, Eva Johanna Kantelhardt, Eric Sven Kroeber, Sefonias Getachew

**Affiliations:** 1 Global and Planetary Health Working Group, Center of Health Sciences, Medical Faculty of Martin-Luther-University Halle-Wittenberg, Halle (Saale), Germany; 2 Department of Epidemiology and Biostatistics, School of Public Health, College of Health Sciences, Addis Ababa University, Addis Ababa, Ethiopia; 3 Department of Gynecology, Medical Faculty of Martin-Luther-University Halle-Wittenberg, Halle (Saale), Germany; 4 Department of Epidemiology and Biostatistics, School of Public Health, College of Medicine and Health Science, Mizan-Tepi University, Mizan-Teferi, Ethiopia; 5 Department of Surgery, College of Health Sciences, Addis Ababa University, Addis Ababa, Ethiopia; 6 Department of Public Health, College of Health Science, Debre Tabor University, Debre Tabor, Ethiopia

**Keywords:** Breast abnormalities, E-health, Referral, Follow-up, Early detection

## Abstract

**Background:**

A weak referral system combined with limited diagnostic facilities is among the key systemic barriers to the early detection of breast cancer. Strengthening patient pathways is essential to improve follow-up and reduce delays in cancer diagnosis and care. This study aims to assess the effectiveness of the DINKNESH referral and follow-up app, a digital, app-based patient referral system linking early detection of breast-related abnormalities at primary healthcare settings with diagnostic facilities in Ethiopia.

**Methods:**

A two-arm cluster randomised trial with an embedded qualitative study is being conducted at eight primary health facilities and affiliated satellite hospitals in Ethiopia. The study includes women aged ≥18 years presenting with breast abnormalities, as well as women aged ≥30 years with positive findings on clinical breast examination. In intervention cluster facilities, the referral process for further diagnosis is supported by the DINKNESH referral and follow-up app, which facilitates patient registration, data transfer, and reminder services. This is compared with the routine paper-based referral process in the control clusters. The primary outcome is the proportion of completed referrals. All outcome measures will be analysed using IBM SPSS Statistics 25.0. A mixed-effects logistic regression model will be applied, adjusting for potential confounders and accounting for clustering at the facility level. At the end of the intervention period, qualitative interviews will be conducted using the RE-AIM framework to explore the acceptability, challenges, sustainability, and scalability of the intervention.

**Discussion:**

This study will provide robust evidence on whether app-based referral systems for women with breast symptoms can improve follow-up and facilitate early breast cancer detection in low-resource settings such as Ethiopia. The findings will support the WHO Global Breast Cancer Initiative’s goal of diagnosing more than 60% of breast cancer cases at an early stage.

**Trial registration:**

PACTR202411893209747. Registered on 25 November 2024, https://pactr.samrc.ac.za/

## Administrative information

Note: The numbers in curly brackets throughout this protocol refer to SPIRIT checklist item numbers. The order of the items has been modified to group similar content areas (see http://www.equator-network.org/reporting-guidelines/spirit-2013-statement-defining-standard-protocol-items-for-clinical-trials/).

**Table:** 

Title {1}	E-health–supported referral for patients with breast abnormalities at primary healthcare facilities in Ethiopia: protocol for a cluster-randomised controlled trial
Trial registration {2a and 2b}	Trial registration: PACTR202411893209747
Protocol version {3}	Version 2
Funding {4}	This study was supported through a grant from the Hospital Partnership, via the Deutsche Gesellschaft für Internationale Zusammenarbeit (GIZ), funded by the German Federal Ministry for Economic Cooperation and Development (ID 81281915). Additional support was provided by the Else Kröner-Fresenius Foundation (Grant No. 2018_HA31SP). The project on which this publication is based was partially funded by the German Federal Ministry of Research, Technology and Space (BMFTR), grant number 01KA2220B, to the RHISSA Programme for the NORA Consortium. This research was also funded, in whole or in part, by the Science for Africa Foundation through the Developing Excellence in Leadership, Training and Science in Africa (DELTAS Africa) programme [Del-22-008], with support from the Wellcome Trust and the UK Foreign, Commonwealth & Development Office. It forms part of the EDCTP2 programme, supported by the European Union.
Author details {5a}	Eyerusalem Getachew: Global and Planetary Health Working Group, Center of Health Sciences, Medical Faculty of Martin-Luther-University Halle-Wittenberg, Halle (Saale), Germany. Department of Epidemiology and Biostatistics, School of Public Health, College of Health Sciences, Addis Ababa University, Addis Ababa, Ethiopia Muluken Gizaw: Department of Epidemiology and Biostatistics, School of Public Health, College of Health Sciences, Addis Ababa University, Addis Ababa, Ethiopia. Global and Planetary Health Working Group, Center of Health Sciences, Medical Faculty of Martin-Luther-University Halle-Wittenberg, Halle (Saale), Germany Endale Anberber: Department of Surgery, College of Health Sciences, Addis Ababa University, Addis Ababa, Ethiopia Abel Shita: Global and Planetary Health Working Group, Center of Health Sciences, Medical Faculty of Martin-Luther-University Halle-Wittenberg, Halle (Saale), Germany Alemnew Destaw: Global and Planetary Health Working Group, Center of Health Sciences, Medical Faculty of Martin-Luther-University Halle-Wittenberg, Halle (Saale), Germany. Department of Epidemiology and Biostatistics, School of Public Health, College of Health Sciences, Addis Ababa University, Addis Ababa, Ethiopia. Department of Epidemiology and Biostatistics, School of Public Health, College of Medicine and Health Science, Mizan-Tepi University, Mizan-Teferi, Ethiopia Sarah Sophie Rossner: Global and Planetary Health Working Group, Center of Health Sciences, Medical Faculty of Martin-Luther-University Halle-Wittenberg, Halle (Saale), Germany Aragaw Tesfaw: Global and Planetary Health Working Group, Center of Health Sciences, Medical Faculty of Martin-Luther-University Halle-Wittenberg, Halle (Saale), Germany. Department of Epidemiology and Biostatistics, School of Public Health, College of Health Sciences, Addis Ababa University, Addis Ababa, Ethiopia. Department of Public Health, College of Health Science, Debre Tabor University, Debre Tabor, Ethiopia Adamu Addissie: Department of Epidemiology and Biostatistics, School of Public Health, College of Health Sciences, Addis Ababa University, Addis Ababa, Ethiopia. Global and Planetary Health Working Group, Center of Health Sciences, Medical Faculty of Martin-Luther-University Halle-Wittenberg, Halle (Saale), Germany Eva Johanna Kantelhardt: Global and Planetary Health Working Group, Center of Health Sciences, Medical Faculty of Martin-Luther-University Halle-Wittenberg, Halle (Saale), Germany. Department of Gynecology, Medical Faculty of Martin-Luther-University Halle-Wittenberg, Halle (Saale), Germany Eric Sven Kroeber: Global and Planetary Health Working Group, Center of Health Sciences, Medical Faculty of Martin-Luther-University Halle-Wittenberg, Halle (Saale) Sefonias Getachew: Department of Epidemiology and Biostatistics, School of Public Health, College of Health Sciences, Addis Ababa University, Addis Ababa, Ethiopia. Global and Planetary Health Working Group, Center of Health Sciences, Medical Faculty of Martin-Luther-University Halle-Wittenberg, Halle (Saale), Germany
Name and contact information for the trial sponsor {5b}	Martin Luther University Halle, Wittenberg, Magdeburger Straße 8, 06112 Halle (Saale) Email: eva.kantelhardt@uk-halle.de Addis Ababa University, Department of Epidemiology and Biostatistics, School of Public Health, Ethiopia
Role of sponsor {5c}	The study sponsor oversees protocol development and ethical clearance, ensuring that the trial adheres to ethical standards and maintains scientific integrity. The funder provides resources, monitors trial progress in line with the approved budget and timeline, and offers feedback as needed.

## Introduction

### Background and rationale {6a}

With an estimated 2.3 million new cases, female breast cancer is the most commonly diagnosed cancer worldwide [1]. Between the mid-1990s and mid-2010s, breast cancer incidence rates in sub-Saharan Africa increased by more than 5% annually, accompanied by high mortality rates in these regions [2]. In low- and middle-income countries, many women are diagnosed at later stages [3].

In Ethiopia, breast cancer is one of the leading causes of cancer-related death, accounting for an estimated 9061 (22.6%) deaths annually [1]. A systematic review revealed that the rate of early breast cancer diagnosis stands at 34.2%, which is far below the 60% target set by the Global Breast Cancer Initiative (GBCI) [4, 5]. A similar study conducted across 17 sub-Saharan African countries, including Ethiopia, reported a median rate of late presentation of 74.7% [6].

Health system-related barriers to early detection of breast cancer in Ethiopia include frequent misdiagnosis at initial contact, long waiting times for diagnostic tests, limited oncological services and infrastructure across healthcare levels, and a weak referral system [7–9].

Ethiopia’s health system is structured in three tiers. The primary level includes primary hospitals, health centres, and health posts. The secondary level comprises general hospitals, which serve as referral facilities for primary hospitals. The tertiary level consists of specialised referral hospitals. Primary healthcare settings are the first point of contact for patients and play a crucial role in both disease prevention and treatment [10].

There is an absence of clear communication protocols and a lack of a formal referral linkage system in Ethiopia [9]. A study in Tanzania revealed that among referred patients with breast abnormalities who underwent a clinical breast examination (CBE), only 52% had a completed referral [11]. A study in Mexico found that women often did not proceed to the next point of referral because of a lack of access to care, an issue compounded by a referral process described by the women as exhausting [12].

For a successful continuum of cancer care, early detection of abnormalities must be linked to timely confirmed diagnosis and prompt treatment. An effective and efficient referral system requires coordination, communication, and feedback mechanisms at all levels, supported by an integrated information recording system [13].

Recent innovations in e-health, such as the digitalisation of patient registries, have improved referral efficiency in underserved communities [14]. E-referrals enhance communication between primary care providers and specialists, increasing the quality of referrals while minimising unnecessary ones. Studies also show that they reduce missing or incomplete information, thereby streamlining the referral process [15, 16].

Given the burden of breast cancer, low socioeconomic status, limited infrastructure, scarcity of advanced cancer care facilities, and weak health system in Ethiopia, there is a need to develop and test contextually appropriate and feasible interventions. These should aim to improve early detection by identifying breast-related abnormalities and ensuring coordinated referrals at the primary healthcare level. This study aims to use the DINKNESH referral and follow-up app within primary healthcare to facilitate referral pathways for women undergoing a CBE, as well as to evaluate the effects of this intervention on improving referral pathways and the early detection of breast abnormalities, including cancer. The evidence gathered will support policymakers in integrating electronic approaches into cancer care and may serve as a model for addressing other health concerns.

## Objectives {7}

The primary objective of this study is to evaluate the effectiveness of the DINKNESH referral and follow-up app in enhancing referral completion for women with breast-related abnormalities at primary healthcare settings in Ethiopia, specifically by determining the proportion of successfully completed referrals.

Hypothesis: The use of the DINKNESH referral and follow-up app will increase the proportion of completed referrals from 52% to 70% for women with breast-related abnormalities after undergoing a CBE compared with the routine standard referral process.

## Trial design {8}

**Fig. 1:**
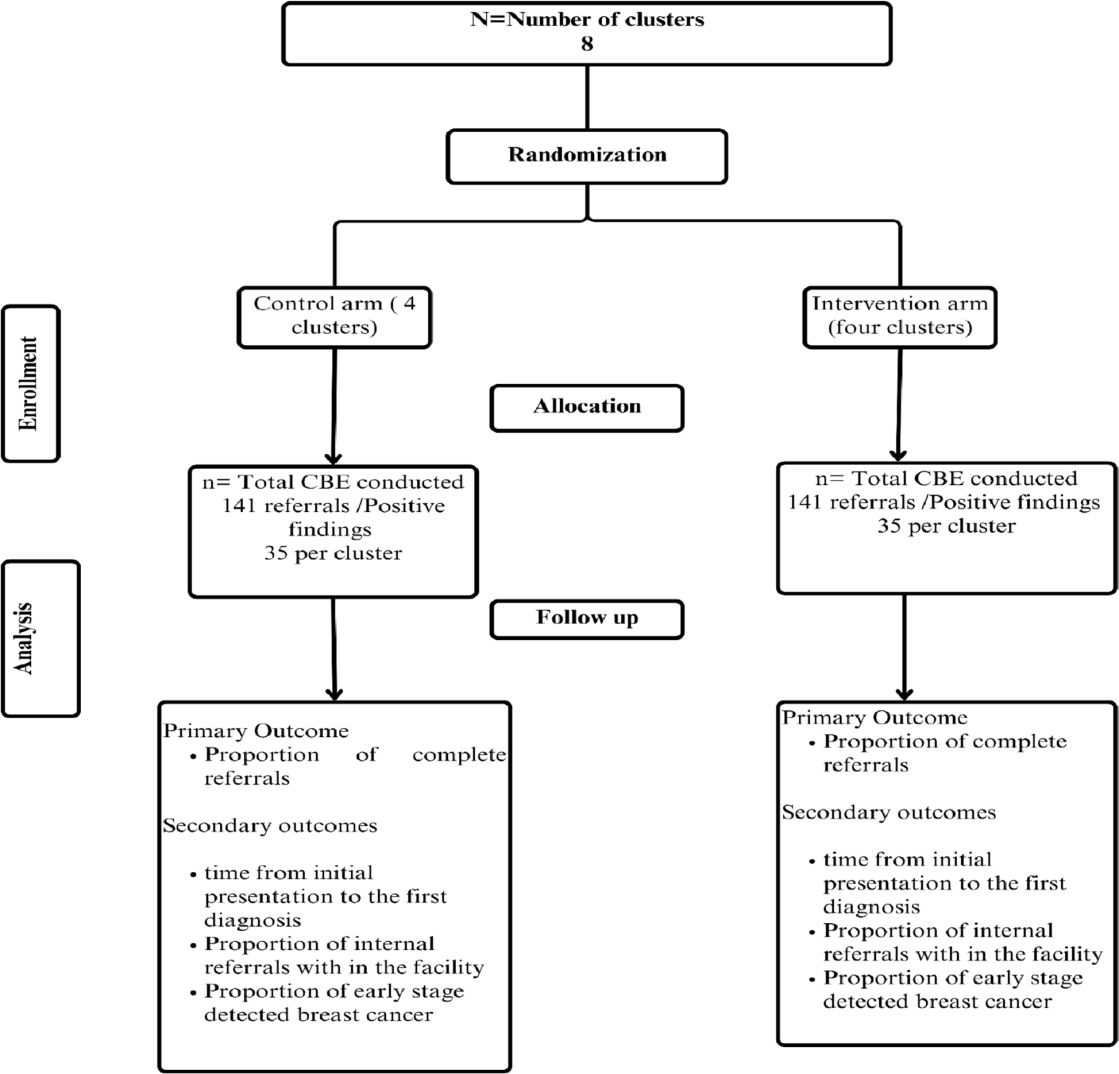
CONSORT diagram of e-health–supported referral of patients with breast abnormalities at primary healthcare facilities in Ethiopia

This is a parallel, two-arm, superiority cluster randomised controlled trial with an embedded qualitative study. The study includes two arms: an intervention arm and a routine standard care arm (Fig. 1).

## Methods: participants, interventions, and outcomes

### Study setting {9}

The study is being conducted at eight primary healthcare facilities (clusters) across three regions of Ethiopia: Oromia, Central Ethiopia, and South Ethiopia and their associated referral hospitals. These facilities were deliberately selected based on prior research and background data because they offer adequate early detection capacity and provide a solid foundation for this interventional study [7, 17, 18]. Each primary healthcare facility is linked to a referral hospital for onward patient referrals.

### Eligibility criteria {10}

Health facilities included in the study are part of the oncology research network supported by the Else Kröner Cancer Center and the DINKNESH Project. Facilities were selected based on their established referral linkages with satellite hospitals. These project sites offer cervical and breast cancer screening, diagnostic, and treatment services.

#### Inclusion criteria

- Women aged ≥18 years presenting with a self-reported breast-related abnormality
- Women aged ≥30 years visiting the health centre outpatient department who are willing to undergo CBE and have an abnormal clinical finding (based on the recent Ethiopian breast cancer guideline [19])

#### Exclusion criteria

- Women who will not undergo CBE
- Men (because of the low number of such patients in the study setting)

### Who will take informed consent? {26a}

Trained data collectors are responsible for obtaining informed consent for trial participation. Women who can read and write will provide written consent themselves. In cases where women cannot read or write, a witness will sign the consent form on their behalf to confirm their participation in the trial.

### Additional consent provisions for collection and use of participant data and biological specimens {26b}

Not applicable: This study does not involve the collection or use of biological specimens.

## Interventions

### Explanation for the choice of comparators {6b}

The control arm represents routine standard care. This involves women presenting at health facilities with or without breast-related abnormalities. A health professional trained in CBE will examine the woman and perform the CBE. Based on the findings, the professional will either recommend treatment or initiate a referral for further management. The woman will be given a referral paper, which she must take to the next facility. Upon her visit to the receiving facility, feedback will be sent back to the referring facility.

### Intervention description {11a}

Intervention/DINKNESH referral and follow-up app:

The proposed intervention in this study is the implementation of a digital, app-based referral and follow-up system called the DINKNESH referral and follow-up app—at primary care settings in peripheral regions of Ethiopia.

#### Intervention development

Following a review of the literature, situational assessments, previous studies at the selected sites, and stakeholder discussions, the principal investigator (E.G.) and the research team analysed the patient care pathway and developed an algorithm for the DINKNESH referral and follow-up app [20]. Key variables to be documented using the app were identified through literature reviews, expert consultation, and consultative meetings held in close collaboration with the Ethiopian Ministry of Health and regional focal persons for cancer and non-communicable diseases. The app developer, IWKET Plc., provided technical support by creating the mobile application based on the algorithm and selected variables, and continues to offer information technology support as needed.

The development process involved several key stages. Initial stakeholder meetings were held with the Ministry of Health, regional and woreda health offices, service users, and non-governmental organisations experienced in implementing electronic health interventions in Ethiopia. These meetings informed the app’s implementation strategy, drew on lessons from past initiatives, and explored integration into existing systems. A draft prototype was pretested to assess usability, functionality, and potential challenges; necessary revisions were made accordingly. A final stakeholder meeting confirmed that the app was ready for implementation.

#### How does the intervention (DINKNESH referral and follow-up app) work?

Women with self-reported or newly detected breast abnormalities identified through CBE will be registered in the DINKNESH referral and follow-up app. Health professionals at the study sites will use the DINKNESH referral and follow-up app as a communication hub. The app will electronically record patient information, send appointment reminders to patients, and provide advance notifications to receiving facilities with relevant details. It will facilitate referrals through automated messages and continuous information exchange.

### Criteria for discontinuing or modifying allocated interventions {11b}

Because the DINKNESH referral and follow-up app intervention poses minimal to low risk, it may be discontinued at the request of the participant. In the event of discontinuation, the reason will be documented.

### Strategies to improve adherence to interventions {11c}

Several strategies are being implemented to ensure sufficient patient flow and high-quality health services. Given the nature of the study, these strategies aim to increase the number of women with breast abnormalities who seek care at health facilities and are referred, allowing for evaluation of the app’s effectiveness in facilitating complete referrals. Activities include strengthening health professionals’ ability to correctly advise women with breast-related abnormalities and make appropriate referrals according to the recommended guidelines. Training is provided on the CBE, implementation of the DINKNESH referral and follow-up app, community sensitisation, and basic orientation for health extension workers. Health extension workers at the study sites receive orientation on breast cancer and breast cancer screening. During their routine household visits, they inform community members about the availability of CBE services at the health centre.

### Relevant concomitant care permitted or prohibited during the trial {11d}

Standard routine primary healthcare, including CBE and routine referrals, is permitted. However, the use of alternative digital referral systems and any additional referral-focused interventions outside of routine care is prohibited.

### Provisions for post-trial care {30}

Following the trial, the control group will receive training on the DINKNESH referral and follow-up app after any necessary modifications have been made based on the trial findings.

### Outcomes {12}

Primary outcome: Proportion of successfully completed referrals.

Definition of primary outcome: A successfully completed referral is defined as a case in which, after study inclusion, a patient initiates the first diagnostic or treatment procedure at the receiving external institution and feedback is received by the referring institution within 60 days of the referral.

Secondary outcomes:

- Time from initial presentation at the health facility to first diagnosis for women with breast-related abnormalities
- Proportion of internal referrals within the facility
- Proportion of breast cancers detected at an early stage

### Participant timeline {13}

Following randomisation, eligible participants visiting the study health facilities are being enrolled (Table 1). In the intervention arm, referral details are being registered and tracked through the DINKNESH referral and follow-up app. Feedback is being recorded upon the patient’s arrival at the referral facility, marking the end of the participant’s study period. In the control arm, referrals are following routine care procedures, and medical records are being reviewed 60 days after referral to assess diagnosis and feedback. The 60-day cutoff for each study participant is based on GBCI recommendations for timely diagnosis [5].

**Table 1:**
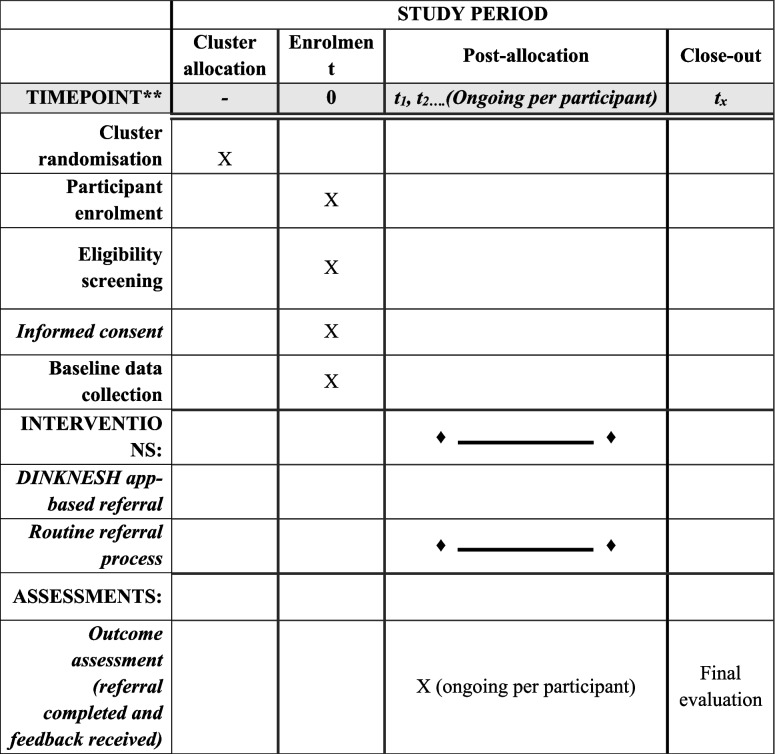
Study timeline for e-health–supported referral of breast abnormalities at primary healthcare facilities in Ethiopia: cluster randomised controlled trial protocol

X: tasks will be completed

### Sample size {14}

The sample size was calculated following established methods for cluster randomised trials [21]. The calculation aimed to detect an increase in the proportion of complete referrals from 52% (based on a study in Tanzania [22]) to 70% in the intervention group. Using 80% power and a 5% significance level, and assuming an intra-cluster correlation coefficient of 0.01, the sample size was adjusted for clustering. With 8 clusters (4 intervention and 4 control) and equal allocation, the required sample size under individual randomisation was 222. After adjusting for the design effect due to clustering, the total required sample size increased to 282, corresponding to approximately 35 individuals per cluster.

### Recruitment {15}

All women with self-reported or CBE-detected breast abnormalities are being invited by trained staff at the health facilities to voluntarily participate in the study. Enrolment will continue until the required sample size of 35 participants is reached at each clustered facility. Once the target number is achieved, no additional participants will be included.

## Assignment of interventions: allocation

### Sequence generation {16a}

To ensure balance between the intervention and control groups, randomisation was stratified by health facility type. Among the eight clusters, two cluster health facilities are primary hospitals; therefore, these two primary hospitals were randomised first. The remaining six health centres were then randomly allocated to the intervention and control groups. The random allocation was conducted by an independent statistician using the lottery method.

### Concealment mechanism {16b}

A lottery method was used to cluster-randomise health facilities into either the control group or the intervention arm, ensuring an unbiased distribution of facilities across the two groups.

### Implementation {16c}

The DINKNESH referral and follow-up app is hosted on a secure server at Addis Ababa University. Sixteen health professionals, two per facility, have been trained and will be responsible for using the app to manage referrals based on eligibility criteria and their clinical decisions. Tablets with the DINKNESH referral and follow-up app installed will be provided to the trained health professionals at each facility. At the primary healthcare facilities, the tablets will be given to professionals working in the outpatient department. At the referral hospitals, nurses working in the surgical outpatient department will be responsible for using the app to receive referrals.

## Assignment of interventions: blinding

### Who will be blinded {17a}

Not applicable: Because this is a cluster randomised controlled trial with the intervention implemented at the health system level, blinding is not feasible.

### Procedure for unblinding if needed {17b}

Not applicable: Blinding will not be implemented in this study.

## Data collection and management

### Plans for assessment and collection of outcomes {18a}

#### Primary outcome (proportion of complete referrals)

For the intervention group, the referral status will be digitally recorded. Time intervals from the woman’s initial visit to the facility to when the referral is sent and when feedback is received will be used to calculate the proportion of complete referrals. In the control group, referrals will be managed through routine paper-based procedures. Sixty days after the referral, trained data collectors will review medical records at both the sending and receiving facilities to determine whether feedback was received. The use of a standardised data extraction tool, aligned with the variables used in the DINKNESH referral and follow-up app, will ensure comparability between the two groups.

To further explain the outcome variable, baseline data will be collected from both groups after consent, including sociodemographic information, clinical data, and knowledge of breast cancer. The African Women Awareness of CANcer (AWACAN) tool will be used to assess awareness, health-seeking behaviour, and perceptions. This baseline information will help interpret the primary outcome [23].

#### Secondary outcomes

The time interval between a woman’s first visit to a health facility and her confirmed breast diagnosis, the proportion of internal referrals within the facility, and the proportion of detected early-stage breast cancers will be assessed. These data will be captured through clinical information recorded in the app and the patient’s medical records. The proportions for each of these secondary outcomes will then be calculated.

#### Qualitative assessment

At the end of the trial, after final follow-up data have been collected for the last referred participant, qualitative assessments will be conducted with participants from the intervention arm and health professionals. Semi-structured interviews will explore the experiences of participants and health providers with the DINKNESH referral and follow-up app, including factors influencing its effectiveness, acceptability, sustainability, and scalability. Interviews will be conducted in Amharic with participants, health professionals, and health facility administrators involved in the intervention. The primary aim is to assess the app’s acceptability, identify implementation challenges, and evaluate its potential for sustainability. Thirty interviews will be conducted, with the final sample size determined by data saturation.

### Plans to promote participant retention and complete follow-up {18b}

In this study, retention is conceptualised as compliance with referral recommendations, which is the key outcome of interest. The strategy to maintain adherence focuses on ensuring that participants receive reminder text messages and that facilities receive notifications and patient information without interruption. To support this, health professionals will register more than one phone number for each participant, including the number of a health extension worker. Reminder text messages will be sent to all registered phone numbers. If a participant does not have her own phone, the number of a close relative registered with her consent will be used in addition to the health extension worker’s number. Regular monitoring will be conducted to identify and address any technical issues that may arise in communication between facilities, such as app malfunctions, network interruptions, or data synchronisation problems.

### Data management {19}

To ensure the quality of the data, data collectors and supervisors were trained prior to implementation. Supervisors are overseeing the implementation of the intervention and proper data documentation. A standard operating procedure (SOP) was developed, and all personnel follow it throughout the study. Both qualitative and quantitative data collection tools were pretested. Baseline information is being collected electronically using Research Electronic Data Capture (REDCap) [24]. For the qualitative data, all recorded audio files will be deleted once transcription is finalised. The translated and transcribed data will be stored confidentially and will be accessible only to the study team.

### Confidentiality {27}

All trial-related data including patient demographic information, diagnoses, investigation results, and treatments provided will be stored on password-protected devices. All paper-based records will be kept in locked cabinets. Patient identifiers will be coded, and the code key will be stored separately to ensure confidentiality.

### Plans for collection, laboratory evaluation, and storage of biological specimens for genetic or molecular analysis in this trial or for future use {33}

Not applicable: The study does not include plans to collect or use biological specimens.

## Statistical methods

### Statistical methods for primary and secondary outcomes {20a}

#### Primary outcome (proportion of complete referrals)

All outcome measures will be analysed using IBM SPSS Statistics 25.0. Baseline differences between the intervention and control groups will be assessed using the independent samples *t*-test for continuous variables and the chi-square (χ^2^) test for categorical variables. To assess the effect of the intervention on the proportion of successfully completed referrals, a mixed-effects logistic regression model will be used, adjusting for potential confounders and accounting for clustering at the health facility level. Results will be reported in accordance with the CONSORT guidelines for reporting clinical trials.

#### Secondary outcomes

Time to diagnosis: The time interval between a woman’s first visit to a health facility and her confirmed breast diagnosis will be measured using clinical records from the app (intervention group) and patient medical records (control group). Descriptive statistics (mean, median, interquartile range, and standard deviation) will be used to summarise the time intervals in both groups. Time to diagnosis will be dichotomised as ≤60 days timely versus >60 days delayed and analysed using multivariable mixed-effects logistic regression adjusting for potential confounders and accounting for clustering at the health facility level.

Proportion of internal referrals and proportion of early-stage breast cancer diagnoses: Descriptive statistics (percentages) will be used to summarise these proportions. Comparisons between the intervention and control arms will be conducted using multivariable mixed-effects logistic regression accounting for clustering at health facility level and adjusting for possible confounders.

#### Qualitative assessment

The audio-recorded interviews will be transcribed and translated into English. Coding of the translated interviews will be conducted using OpenCode software for qualitative data analysis (version 4.03). The RE-AIM framework will guide the analysis of the qualitative data [25]. Coded interviews will be categorised and sub-categorised based on their content. These categories will inform the development of main themes and sub-themes. The findings will help to further explain the impact of the intervention from multiple perspectives.

### Interim analyses {21b}

An interim analysis is not planned because the study poses minimal safety concerns and is being implemented within established healthcare facilities. The control group will continue receiving standard care, which helps minimise immediate safety and ethical concerns. Additionally, a complete dataset is required to properly assess the study outcomes.

### Methods for additional analyses (e.g. subgroup analyses) {20b}

Subgroup analyses will be conducted to explore the effectiveness of the intervention in relation to participants’ sociodemographic status, knowledge, and health-seeking behaviour, thus helping to further explain the primary outcome variable.

### Methods in analysis to handle protocol non-adherence and any statistical methods to handle missing data {20c}

To minimise missing data, data collectors will be trained on the standard operating procedure for data collection. Strong supervision and regular follow-up will be implemented throughout the study. In cases of protocol non-adherence or missing data, an intention-to-treat analysis will be used, in which all participants enrolled at the start of the study will be included in the analysis. The approach to data analysis will depend on the type of missing data encountered [26]. Additionally, a sensitivity analysis will be conducted to assess the robustness of the results under different assumptions about missing data.

### Plans to give access to the full protocol, participant-level data, and statistical code {31c}

Following publication of the study, the analysis code and de-identified data will be made available upon reasonable request to the corresponding author, subject to approval by the ethics committee and the trial supervisory team. The manuscript describing the study protocol will be published in an open-access journal.

## Oversight and monitoring

### Composition of the coordinating centre and trial steering committee {5d}

The study will be overseen by a supervisory team that includes epidemiologists, a statistician, ethicists, clinicians, and the principal investigator. The team will hold weekly meetings to monitor the trial progress and ensure adherence to the study protocol. It will also review potential risks or ethical challenges and make strategic decisions regarding trial implementation when necessary.

### Composition, role, and reporting structure of the data monitoring committee {21a}

In this trial, the programme steering committee represented by the supervisory team determined that an independent data monitoring committee was not necessary. This decision was based on the absence of planned interim analyses, which typically require independent data review and decision-making. However, the supervisory team will continue to monitor for any major study discontinuation events or instances of trial misconduct.

### Adverse event reporting and harms {22}

Any adverse events related to the DINKNESH referral and follow-up app will be documented and reported to both the ethics review board and the study’s supervisory team.

### Frequency and plans for auditing trial conduct {23}

The supervisory team will review the study data at baseline and monitor the overall conduct of the trial. This includes checking data quality, compliance with ethical recommendations, informed consent procedures, and general trial implementation.

### Plans for communicating important protocol amendments to relevant parties (e.g. trial participants, ethical committees) {25}

In the event of any protocol amendments, the Institutional Review Board of Addis Ababa University College of Health Sciences and the Ethics Commission of Martin Luther University will be notified, and approval will be sought from both institutions. Amendments will also be reported to the trial registration platforms, including the World Health Organization’s (WHO’s) Registry Network and the Pan African Clinical Trials Registry (PACTR).

## Dissemination plans {31a}

The findings will be published in peer-reviewed journals and presented at both international and local conferences. In addition, the results will be shared and discussed with relevant stakeholders.

## Discussion

The WHO recommends integrating digital health services to strengthen health systems and advance universal health coverage [27]. In addition, the WHO’s GBCI emphasises early detection and timely referral of suspected breast lesions to ensure early diagnosis [5]. However, evidence on the impact of digital health in primary cancer care—particularly in low-resource settings—remains limited [28]. This study aims to help fill that gap by evaluating a digital referral system designed to support early breast cancer detection and contribute to the GBCI goal of diagnosing 60% of women at an early stage.

The lack of blinding is a limitation of this study because it may introduce bias. Additionally, the relatively short follow-up period of 60 days may restrict understanding of the longer-term referral process following diagnosis. Because the study follow-up focuses primarily on the time to early diagnosis, subsequent referrals for treatment and ongoing care may not be captured.

A key strength of this study is its focus on peripheral health settings, where evidence remains scarce. It also contributes to capacity building by providing CBE training to healthcare providers. Furthermore, the embedded qualitative study will explore the implementation challenges and user experiences associated with the DINKNESH referral and follow-up app, offering valuable insights to inform future scale-up plans. The intervention is expected to increase the proportion of completed referrals and improve the early diagnosis of breast abnormalities by addressing referral delays caused by poor communication between facilities. Moreover, it will provide policymakers with insights into the use of digital referral systems in cancer care, including both challenges and opportunities for broader implementation.

## Trial status

Registered at World Health Organization’s (WHO’s) Registry Network and the Pan African Clinical Trials Registry (PACTR) No. PACTR202411893209747. Enrolment began on 30 November 2024.

## Data Availability

The trial dataset is not publicly available because of the inclusion of detailed patient information. However, it may be made available upon reasonable request to the principal investigator, subject to approval by the relevant ethics committee.

## Abbreviations

AWACAN: African Women Awareness of CANcer
CBE: Clinical Breast Examination
DINKNESH: Developing an InterNational research collaboration in Ethiopia is under implementation to support oncology at primary Health care levels
GBCI: Global Breast Cancer Initiative
PACTR: Pan African Clinical Trials Registry
WHO: World Health Organization
